# Nurturing Global Leadership, Advocacy, Research, and Collegiality: The Unique Experience of The International Society of Nephrology Emerging Leaders Program

**DOI:** 10.1016/j.ekir.2023.07.001

**Published:** 2023-07-13

**Authors:** Sabine Karam, Muhammad Iqbal Abdul Hafidz, Viviane Calice-Silva, Titi Chen, Sophie Dupuis, Udeme E. Ekrikpo, Anna Francis, Vivekanand Jha, Robert Kalyesubula, Vivek Kumar, Georges Nakhoul, Nikhil Pereira-Kamath, Elliot K. Tannor, Anh Tran, Eranga Wijewickrama, Michelle M.Y. Wong, Rahul Chanchlani

**Affiliations:** 1 Division of Nephrology and Hypertension, University of Minnesota, Minneapolis, USA; 2 Department of Nephrology, Universiti Teknologi MARA, Selangor, Malaysia; 3 Pro-Rim Foundation, Joinville, Santa Catarina, Brazil and Department of Clinical Medicine, Faculty of Medicine, University of the Region of Joinville (UNIVILLE), Joinville, Santa Catarina, Brazil; 4 Department of Renal Medicine, Westmead Hospital, Westmead, New South Wales, Australia; 5 International Society of Nephrology, Brussels, Belgium; 6 Department of Medicine, University of Uyo, Uyo, Nigeria; 7 Department of Clinical Medicine, School of Medicine, University of Queensland, Brisbane, Australia; 8 George Institute for Global Health, UNSW, New Delhi, India; 9 School of Public Health, Imperial College, London, UK; 10 Department of Physiology, Makerere University College of Health Sciences, Kampala, Uganda; 11 Department of Nephrology, Postgraduate Institute of Medical Education and Research, Chandigarh, India; 12 Department of Nephrology and Hypertension, Cleveland Clinic, Cleveland, Ohio, USA; 13 Africa Healthcare Network Tanzania Limited, Dar es Salaam, Tanzania; 14 Department of Medicine, Kwame Nkrumah University of Science and Technology, Kumasi, Ghana; 15 Department of Clinical Medicine, Faculty of Medicine, University of Colombo, Colombo, Sri Lanka; 16 Division of Nephrology, Department of Medicine, University of British Columbia, Vancouver, British Columbia, Canada; 17 Division of Pediatric Nephrology, Department of Pediatrics, McMaster Children’s Hospital, Hamilton, Ontario, Canada

### Introduction

In 2020, coinciding with its 60th anniversary, the International Society of Nephrology (ISN) launched its Emerging Leaders Program (ELP), the first of its kind by a major nephrology society. The program was conceived with the intention to build a global cadre of nephrology leaders who would strive together for a future where all people would have an equitable access to sustainable and quality kidney health care, in alignment with the mission and vision of the ISN. This can be achieved by providing the opportunity to partake in ISN’s global capacity-building programs and workgroups, and work closely with international experts to develop skills in implementation science and research. The program also aims to enhance capacity at developing and implementing context-specific, resource-sensitive, evidence-based, multidisciplinary care to those with and at-risk of kidney diseases. In addition, the focus is on acquiring the necessary skills in the evaluation, development, and management of health systems along with principles of entrepreneurship, advocacy, and communication to ensure better equity and diversity. Finally, the program provides access to continuous professional development to take on leadership roles locally and globally.

A call for expression of interest was sent to early career ISN members, defined as those who have been less than 10 years out of professional training. Candidates were asked to submit a portfolio detailing their professional, scholarly, and academic activities to date but most importantly to identify issues related to kidney health that they planned to address through collaborative studies within the program. Of the 71 applications representing all 10 ISN geographical regions, the ELP Steering Committee selected a total of 14 candidates from 12 countries (6 ISN regions). In terms of gender distribution and disciplines, 5 were women, 11 were adult nephrologists, 2 were pediatric nephrologists along with a non-physician entrepreneur, who had established a dialysis provider network in East Africa.

### The First Cohort’s Journey: Webinars, Courses, Workshops, Integration Into ISN Workgroups, Participation in the World Congress of Nephrology, and Implementation Projects

Over the course of 12 months, we attended webinars, courses, and workshops aimed at nurturing our understanding of global nephrology challenges, building capacity in leadership and communication, and leveraging advocacy and partnership skills. We met with major ISN leaders who have forged the organization’s identity, and with major global health actors from the World Health Organization and the Noncommunicable Disease Alliance. In addition, we were invited to work with various ISN working groups. This provided us with deep insights into various ISN core programs such as the design of the ISN Academy; the coordination and design of networks of clinical trials; and the development of fellowship, mentorship, and advocacy programs and structures. To nurture further communication and advocacy skills, we were solicited to moderate topical networking sessions during the last 2 editions of World Congress of Nephrology, which centered around a variety of topics such as chronic kidney disease prevention, social justice, and kidney outcomes (Table 1). In addition, we were invited to attend the precongress advocacy and policy course at the World Congress of Nephrology and learned the principles and practicalities of designing and conducting effective advocacy campaigns. Currently, 3 of our members sit on the ISN Advocacy Working Group representing their regions. Finally, our capacity to self-reflect was developed through a personal effectiveness workshop that allowed us to analyze our personal attributes, better understand ourselves, and improve our leadership skills.

**Table 1:** Summary of Spotlight sessions and Table Talks moderated by Cohort 1 at WCN’23

Spotlight sessions and table talks	Key conclusions	Actionable plan
CKD and Primary Health Care workers: Strategies to narrow down gaps and increase diagnosis	Integrated CKD care in the primary healthcare system needs to start with improvements in awareness and development of strategies to increase CKD detection by the primary health care workers considering models of care which suits on each setting.	• Increase CKD representation in NCD programs worldwide. • Develop strategies to improve diagnosis, identification, and adequate management of individuals at high risk of CKD in the future. • Reduce the gaps between the need and the availability of care. • Efforts in prevention.
Post-AKI care: is it neglected/underestimated in practice?	Post-AKI follow up rates generally are quite low worldwide	• Increase awareness of patients and health care providers at all levels about significance of AKI, its potential to cause long-term consequences, and need for regular follow up. • Understand patient and provider level barriers responsible for poor follow up. • Implement sustainable solutions relevant to the local context to improve post-AKI follow up
How can we better communicate with the community and governments about kidney health advocacy initiatives?	We need to understand the rudiments of advocacy as kidney health professionals to ensure effective communication during community engagement and when we get opportunities with key policy stakeholder.	• Learn some basic skills in communication and advocacy to improve advocacy in kidney health • Start from small, organized groups and social media, and graduate to radio and television to reach the community, region, and country. • Reach out to other health care professionals who have been successful advocates to get some experience.
This is water: is work-life balance possible in Nephrology?	Numerous challenges remain especially in low resource settings	• Balancing personal/family and professional duties. • Acknowledge the importance of mental health for an optimal work environment and address any barriers. • Have institutional recreational goals.
Is it time for teachers in Nephrology to think just beyond teaching?	Traditional teaching curricula and ways have not kept pace with changing needs and objectives of profession	• Innovate to adopt new technology. • Encourage to think beyond just assimilation of knowledge. • Inculcate spirit of teamwork and collaboration in students. • Broaden horizon from just descriptions of diseases and their treatment.
Do nephrologists have a role in community engagements for education in CKD prevention, especially in LMICs?	Nephrologists are few in LMICs and need to devise task-shifting or task-sharing innovations for public education and engagement in CKD prevention.	• Advocate for training of more nephrologists • Optimize the use of remote technology • Train other cadres in key messages and task shift/share screening and education to other cadres in health.

AKI, acute kidney injury; CKD, chronic kidney disease; LMICs, low- and middle-income countries; NCD, Noncommunicable Disease Alliance; WCN, World Congress of Nephrology.

A unique feature of the program was the opportunity to work on collaborative projects impacting the kidney health agenda worldwide. We were first invited to reflect on the access to essential medicines in nephrology. To that effect, we developed and disseminated an international survey evaluating barriers to accessing essential medicines in low-income and low-middle income countries for people with chronic kidney disease. Our results that were published in *Kidney International Reports*, demonstrated poorer access to all essential kidney medicines in community settings compared with tertiary settings.^1^ Community settings were also more likely to report barriers of lack of resources and logistics; whereas barriers related to national health policy, such as scarce government health care funding, were uniform across all health care settings. Currently, we are engaged in a scoping review aimed at evaluating access to essential medicines as well as barriers and interventions aimed to improve access.^2^ In addition, we were introduced to the topic of implementation research through a series of presentations by experts and were offered formal training through an online course. We were subsequently invited to design concrete projects to improve care among patients with kidney diseases. To conceptualize our ideas, we created workgroups for specific projects that reported to the whole group to reflect and receive feedback from fellow ELP members and the ELP steering committee. Our goal is to develop practical and locally appropriate models of care by integrating practices to improve kidney care at an organizational-level or at the systemic-level, while leveraging local experience and resources and facilitating collaboration with primary care providers. One of our projects will aim to improve the rate of chronic kidney disease identification and treatment among high-risk subjects in primary care in low-income and middle-income settings. Another project will assess the effect of implementing a patient-centric and provider-centric post-acute kidney disease discharge communication strategy to improve follow-up in diverse settings in India, Brazil, Canada, Uganda, and Sri Lanka. In addition to engaging with organizational and government leadership, we have designed a qualitative study to obtain perspectives from patients, health care providers, and other stakeholders, to understand the local contextual factors, such as barriers and facilitators to implementing evidence-based innovations.^3^ This process will inform the design of the interventions and our implementation evaluation strategy. Our subsequent pilot projects will have a hybrid-design methodology that assesses implementation outcomes, such as adoption, reach, fidelity, and maintenance of the intervention, while observing effectiveness in improving care processes and patient outcomes. After receiving the required funding and performing pilot studies, we aim to conduct future research to adapt the interventions to multiple settings and test their effectiveness on a broader scale.

### Skills Gained, Impact Achieved, and Lessons Learned

The ELP program has had an invaluable impact on our personal and professional developments as young leaders in nephrology. Many of us, for instance, were given the opportunity to play active roles in international projects such as the Global Kidney Health Atlas, the ISN/DOPPS collaborations with numerous publications,^4^, ^5^, ^6^ the Kidney Disease Improving Global Outcomes Controversies Conference on Improving CKD Quality of Care, and a multisociety cardiorenal initiative developing health care provider toolkits.^7^ In addition, some members had been leading advocacy campaigns in their local regions and shared their experience by collaborating to publish several manuscripts such as an editorial on the role of education and community engagement to improve kidney health for all,^8^ and another one aimed at raising awareness about the threats faced by dialysis patients in the setting of economic crises taking as examples Lebanon and Sri-Lanka.^9^ Moreover, some members of the cohort had opportunities to attend and interact at the World Health Summit where they advocated for the integration of kidney disease at the forefront of the agenda of noncommunicable diseases eradication. Finally, there has been active advocacy work by some members leading nongovernmental organizations that educate and create awareness on hypertension, diabetes, and kidney disease as well as train health care providers in community hospitals in Africa.S1

However, the most valuable impact was widening our individual perspectives. Working with a diverse group of like-minded professionals from all over the world not only exposed us to different healthcare systems, skillsets, cultural specificities, but also allowed us to realize that the barriers faced by patients and health care providers to achieve sustainable kidney care are often universal. We also learned to collaborate with people from different backgrounds and were able to appreciate different outlooks. Listening to and coordinating the views of 14 different leaders was at times challenging, and taught us to know when to assume leadership, and when to let go of it. Another important lesson learned was the necessity at times to redesign ambitious projects at a smaller scale to achieve more realistic goals with the available skills and resources. Indeed, when working at the national or international stages, many barriers can come from actors as large as major governmental organizations. This allowed us to cultivate determination and perseverance and prepared us to better deal with setbacks. Furthermore, the program empowered us to take more active leadership roles in our own organizations and health systems. Most importantly, the program catalyzed the establishment of a unique collaborative network, and of special friendships and sets of skills for many years to come in a distinguished collegial atmosphere and environment (Figure 1).

**Figure 1:**
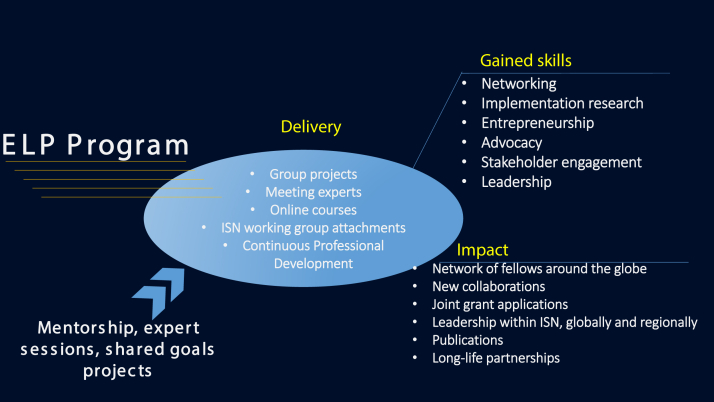
Gained skills and impact of the ELP program.

### A Glance at the Second Cohort

In May 2022, the 12 members of the second ELP cohort began activities on collaborative leadership and problem-solving in global kidney health. Composed of a diverse cohort of nephrologists, pathologists, and researchers, their area of focus is the complex interplay of environmental factors and kidney health, and will be addressed through different angles and projects geared at increasing awareness and implementation of “Green Nephrology”.

### Conclusion: Our Five-Year Vision

The unique ELP of the ISN has provided us a unique experience with involvement that extends far beyond the official duration of the program (Table 2). Members are now playing an active and important role in more than 1 ISN initiative or program. In addition to initiating and pursuing collaborations and mutual reinforcement of skills and capabilities through diverse ISN structures, we have managed to build a unique network of like-minded professionals tightly bound by the same passion for self-development and change. Together, we hope to continue to contribute significantly to ISN’s mission and vision and to pursue our initiation to implementation research by designing and conducting valuable projects in a field that will prove to be essential to the development and sustainability of global kidney health.

**Table 2:** Five-year vision of Cohort 1 of ELP

• Focus on “implementation science” as an important component of global nephrology research by engaging in implementation science training and through the design and conduct of 2 implementation science projects.
• Participation in the elaboration of the research, education, and advocacy portfolios of the ISN through involvement in ISN working groups and/or regional boards.
• Constitution of a unique collaborative network of global leaders working together toward the realization of sustainable kidney health.
• Collaborative projects aimed at improving global kidney care, including assessment of access to essential medicines in nephrology, barriers to access, and solutions to address barriers.
• Create diverse groups of professionals and stakeholders to better understand local problems, propose context-specific solutions and take a lead in implementing such solutions.

ELP, Emerging Leaders Program; ISN, International Society of Nephrology.

## Supplementary Material

Supplemental File (Word)
