# Supplementary material for: Nurturing Global Leadership, Advocacy, Research, and Collegiality: The Unique Experience of The International Society of Nephrology Emerging Leaders Program
Source: Kidney Int Rep. 2023 Jul 13;8(9):1703–7. doi: 10.1016/j.ekir.2023.07.001 (PMC10496062; doi:10.1016/j.ekir.2023.07.001)
Supplement: Supplemental File (Word) [file mmc1.docx]

Supplementary References

S1. Tannor E, Tannor A, Gatorwu S, Osei-Agyemang C. WCN23-0162 promoting’kidney health for all’in Ghana–the role of a nephrology-led non-governmental organization. *Kidney Int Rep*. 2023;8:S465. doi:[10.1016/j.ekir.2023.02.1043](https://doi.org/10.1016/j.ekir.2023.02.1043)
